# TLR2 as a Key Mediator: Bergamottin Protects Against Lead‐Induced Renal Toxicity by Modulating Oxidative Stress and Pyroptosis Pathways

**DOI:** 10.1002/fsn3.71172

**Published:** 2025-11-10

**Authors:** Yanping Zhu, Kaisheng Zhang, Song Wen, Yang Zhang

**Affiliations:** ^1^ Department of Urology Surgery The First Affiliated Hospital, and the College of Clinical Medicine of Henan University of Science and Technology Luoyang China; ^2^ School of Medicine Shaoguan University Shaoguan Guangdong China; ^3^ Department of Oncology Ganzhou Cancer Hospital Ganzhou China

**Keywords:** bergamottin, oxidative stress, Pb, pyroptosis, renal injury, TLR2

## Abstract

Lead (Pb) toxicity is a major health concern, with the kidney being one of the primary target organs. Current therapeutic strategies for Pb‐induced nephrotoxicity are limited, highlighting the need for effective protective agents. Bergamottin (BGM), a natural furanocoumarin, has been reported to possess antioxidant and anti‐inflammatory properties, but its role in Pb‐induced renal injury has not been elucidated. In this study, we investigated the protective effects of BGM against Pb‐induced nephrotoxicity and explored the underlying mechanisms. BGM treatment notably mitigated Pb‐induced renal dysfunction, evidenced by significant reductions in serum creatinine and BUN levels. It also effectively ameliorated oxidative stress by reducing MDA and increasing antioxidant enzyme activities. BGM treatment also decreased the expression of NLRP3, caspase‐1, and IL‐18, thus alleviating pyroptosis‐induced renal injury. Moreover, we found that TLR2 is the key factor; silencing TLR2 enhanced BGM's efficacy in protecting against Pb‐induced renal damage, while overexpressing TLR2 attenuated BGM's protective benefits in HK‐2 cells, highlighting the crucial role of TLR2 in mediating BGM's effects. These findings indicate that BGM could serve as a potential therapeutic agent for Pb‐induced nephrotoxicity, with its protective effects primarily mediated through modulation of TLR2 and subsequent reduction in oxidative stress and pyroptosis.

## Graphical abstract

Bergamottin protects against lead‐induced renal damage by reducing oxidative stress and pyroptosis in C57BL/6 mice. The protective effects of BGM are mediated through TLR2 modulation, as silencing TLR2 enhances its efficacy, while overexpressing TLR2 weakens its protective action in HK‐2 cells. BGM could be a potential therapeutic agent for lead‐induced nephrotoxicity.

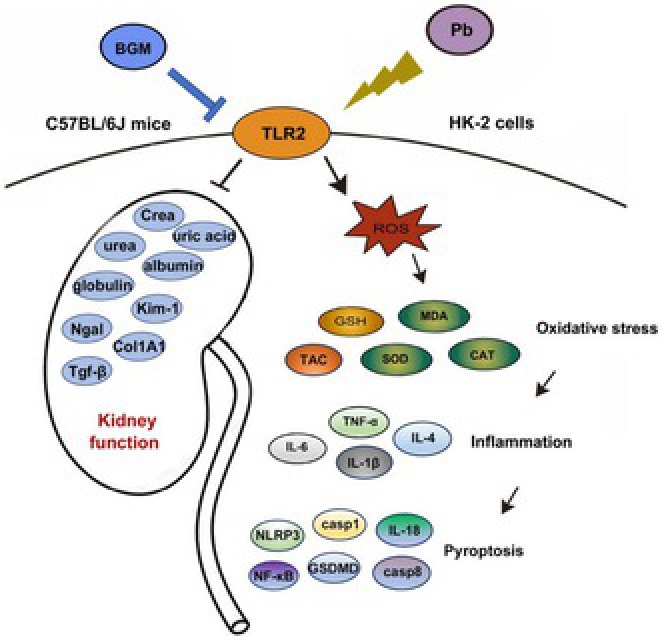

## 1 Introduction

Heavy metals are pervasive environmental and food contaminants, posing significant risks to human health (Mitra et al. 2022). As a heavy metal contaminant, lead (Pb) has garnered considerable attention due to its toxicological effects (Nag and Cummins 2022). Despite global awareness of the dangers of lead exposure, a substantial portion of the population continues to be affected (Gilani et al. 2015). Once in the bloodstream, Pb has a half‐life of approximately 1 month, after which it accumulates in organs such as the kidneys, liver, brain, teeth, and bones (Engwa et al. 2019). The kidneys, in particular, are highly susceptible to Pb‐induced damage due to their role in clearing non‐enzymatic proteins, like thymosin β4, that have a high affinity for lead (Apaydın et al. 2016; Mabrouk et al. 2016). Prolonged exposure to Pb can result in severe kidney damage and compromised renal function (Liu et al. 2017). Given the widespread exposure and the critical health impacts of lead accumulation, understanding its pathological effects, especially on vital organs like the kidneys, is crucial for developing protective strategies.

As an important physiological event, oxidative stress plays an important role in the toxicological damage of Pb, which is the main toxicological mechanism of Pb (Matović et al. 2015). Pb ingestion leads to an excessive accumulation of ROS and a decrease in antioxidant enzyme activity by binding to sulfhydryl groups (‐SH) and forming thiol salts, thereby initiating oxidative stress (Aouini et al. 2018). This imbalance depletes antioxidant defenses, disrupts cellular homeostasis, and promotes tissue damage (Batool et al. 2017). Additionally, oxidative stress is also closely related to events such as inflammation and pyroptosis (Dong et al. 2020; Minutoli et al. 2016), which are important factors in the toxicological damage of environmental pollutants (Mou et al. 2023). Thus, oxidative stress not only drives Pb‐induced kidney injury but also triggers a cascade of secondary damage, such as inflammation and pyroptosis, amplifying the harm caused by Pb. For example, Lv et al. (2023) stated that IRAK1/TAK1/IKK‐mediated pyroptosis and inflammation are pivotal in selenium's protective effects against Pb‐induced CIK cell injury (Lv et al. 2023). Similarly, Wei et al. (2021) identified that ROS/NLRP3/caspase‐1‐mediated pyroptosis plays a significant role in cadmium‐induced apoptosis (Wei et al. 2021). Therefore, reducing oxidative stress and mitigating its downstream effects represent key therapeutic approaches for treating Pb‐induced damage.

In recent years, bioactive compounds from dietary plants have gained attention for their protective effects against damage caused by environmental and food contaminants (Guan et al. 2021). Furanocoumarins, natural bioactive compounds commonly found in foods and cosmetics, are among the most studied (Ahmed et al. 2020; Rodrigues and Rodrigues 2021). Bergamottin (BGM), a furanocoumarin abundant in citrus fruits, has shown potent biological activities, including anti‐inflammatory and antioxidant effects (Maugeri et al. 2021). The potential of plant‐derived compounds such as BGM to mitigate heavy metal‐induced damage highlights a promising natural intervention strategy. BGM has been found to protect against liver injury in cafeteria diet‐fed models by attenuating inflammatory responses and mitigating endoplasmic reticulum and oxidative stress, suggesting its potential as a therapeutic agent for liver diseases (Yazıcı et al. 2023). Additionally, BGM has been reported to slow the progression of osteoarthritis by activating the Sirt1/NF‐κB pathway, thereby inhibiting extracellular matrix degradation and inflammation (Shen et al. 2024). An et al. (2021) further reported that BGM's anti‐inflammatory properties are mediated through the suppression of NF‐κB activation in LPS‐induced lung injury models (An et al. 2021). These studies underscore the multifaceted protective effects of BGM and its potential for broader applications, including in the context of heavy metal toxicity.

Given these promising findings, an in‐depth exploration of the protective mechanisms of BGM is crucial for advancing public health. This study aims to investigate the molecular mechanisms by which BGM alleviates Pb‐induced renal injury. By understanding how BGM modulates oxidative stress, inflammation, and pyroptosis in Pb‐induced kidney damage, we can uncover novel therapeutic approaches to mitigate the harmful effects of lead exposure.

## 2 Materials and Methods

### 2.1 Chemicals

Lead dichloride (PbCl_2_, #YS148319) and bergamottin (BGM, #7380‐40‐7) were sourced from Beijing Solarbio Technology Co. Ltd. The assay kits for SOD, CAT, GSH, MDA, IL‐6, IL‐1β, TNF‐α, IL‐4, creatinine, urea, uric acid, globulin, albumin, and total protein were purchased from Nanjing Jiancheng Co. Ltd.

### 2.2 Animal Experiment

Forty‐eight C57BL/6 (4 weeks old, male, 17–20 g) were purchased from Saiye Biological Company and housed in standard laboratory conditions. After a 3‐week adaptation period, mice were randomly assigned to four groups: control group, Pb group, Pb + L‐BGM (0.05 mg/kg), and Pb + H‐BGM (0.22 mg/kg). The control group received 0.2 mL of saline (0.9% NaCl) via oral gavage. Mice in the Pb group were administered 20 mg/kg PbCl_2_ by gavage, while those in the treatment groups were given PbCl_2_ followed by BGM (0.05 or 0.22 mg/kg) 2 h later, once daily for 10 weeks. The dosages were determined based on previous studies (Cheng et al. 2024; Yazıcı et al. 2023). BGM was first dissolved in dimethyl sulfoxide (DMSO) to obtain a stock solution and then diluted with normal saline to prepare the working solutions at doses of 0.05 mg/kg and 0.22 mg/kg. All experimental protocols involving mice were performed in compliance with the guidelines of the National Institutes of Health and approved by the Animal Ethics Committee of Shaoguan University (No. 8023).

### 2.3 Kidney Function Assessment

Renal function indicators in serum were detected using ELISA kits. Kits of creatinine (#KKX‐56946 M), urea (KKX‐56947 M), uric acid (KKX‐71306 T), albumin (KKX‐56569 M), globulin (KKX‐56923 M), and total protein (KKX‐57286 M) were obtained from Kakaixi Bio. Company (Shanghai, China).

### 2.4 H & E Staining

At the end of the experiment, mice were anesthetized, and their kidneys were excised for histological analysis. H & E staining was performed according to standard protocols as previously described (Qiongyue et al. 2022).

### 2.5 Immunohistochemistry

TLR2 expression in kidney tissue was assessed through immunohistochemistry. Tissue sections were dewaxed, rehydrated, and subjected to antigen retrieval, followed by blocking with serum. Primary antibodies were applied overnight at 4°C, and secondary antibodies were used for detection. The sections were then stained with hematoxylin, dehydrated, and mounted for microscopic evaluation (Kucukler et al. 2021).

### 2.6 qRT‐PCR

Total RNA was isolated from kidney tissue, and its purity was verified. RNA was reverse transcribed into cDNA, and qRT‐PCR was performed using SYBR Green in a CFX thermal cycler (Zhu et al. 2020). Primer sequences used were as follows: NLRP3 (F: GGCTGCTATCTGGAGGAACTT, R: CATCTTCAGCAGCAGCCCTT), p65 (F: CTTCCTCAGCCATGGTACCTCT, R: CAAGTCTTCATCAGCATCAAACTG), GAPDH (F: GGTGAAGGTCGGTGTGAACG, R: CTCGCTCCTGGAAGAATGGTG), caspase‐1 (F: GAGAAACATCCAAAAGTGAGGG, R: GCCTTTCTTCTGGTCAGTGC), caspase‐8 (F: GCTGCCCTCCAAGTTCCTGT, R: GATTGCCTTCCTCCAACATC), GSDMD (F: CCATCGGCCTTTGAGAAAGTG, R: ACACATGAATAACGGGGTTTCC), IL‐18 (F: ATCAGACCACTTTGCAGACTT, R: CTTCCATCCTTCACAGATAGGG).

### 2.7 ELISA Assay

Kidney tissues were homogenized in 0.9% saline using a mechanical homogenizer in an ice‐water bath to prepare a 10% tissue homogenate. Following centrifugation at 2500 rpm for 10 min, the supernatant was collected for analysis of inflammation and oxidative stress markers using ELISA kits for SOD (GM1133), MDA (GM1134), CAT (GM1136), TAC (GM1141), GSH (GM1135), IL‐6 (GEM0001), IL‐1β (GB300002‐M), IL‐4 (GEM0008), and TNF‐α (GEM0004), all purchased from Saiweier Technologies.

### 2.8 Pb Content Determination

Kidney tissue samples weighing 0.2–0.5 g were digested with a mixture of 5 mL nitric acid and 1 mL perchloric acid. The digestion process was either performed at 180°C for 40 min or at 150°C for 4 h using the traditional method until the solution became clear. After digestion, samples were diluted to 25 mL, and lead content was measured using atomic absorption spectrometry (AAS) with a graphite furnace at a wavelength of 283.3 nm.

### 2.9 Statistical Analysis

Data are expressed as mean ± SD. Statistical analyses were performed by GraphPad Prism. One‐way ANOVA was used to compare differences between groups (*p* < 0.05).

## 3 Results

### 3.1 BGM Alleviates Pb‐Caused Kidney Damage in Mice

**FIGURE 1:**
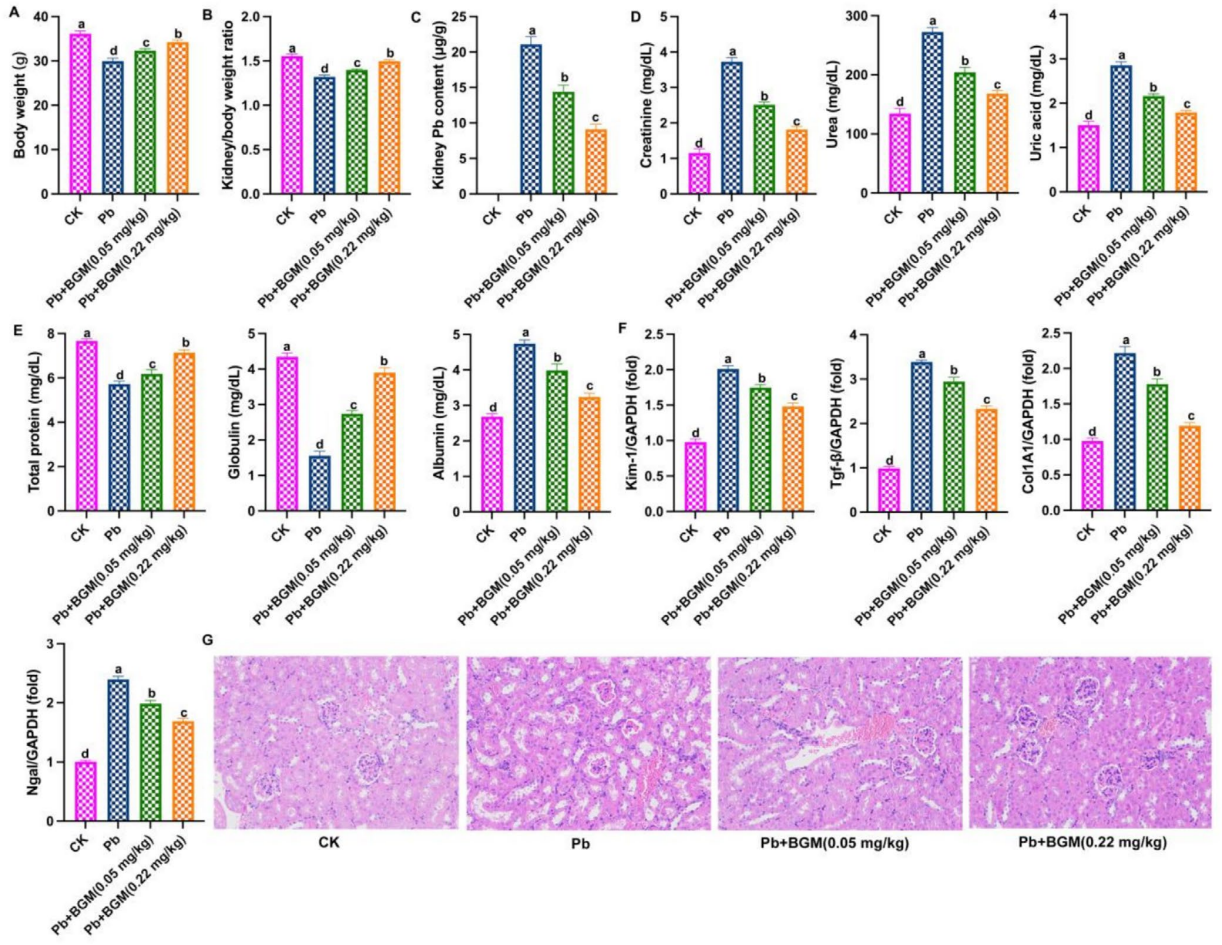
BGM alleviates Pb‐caused kidney damage in mice. (A) Body weight; (B) kidney/body weight ratio; (C) kidney Pb content; (D) levels of creatinine, urea, and uric acid; (E) levels of total protein, albumin level, and globulin level in serum; (F) mRNA expressions of Kim‐1, Ngal, Col1A1, and Tgf‐β. (G) H&E staining. Different letters mean significant differences (*p* < 0.05).

As shown in Figure 1A,B Pb exposure decreased mouse body weight and kidney:body weight ratio, while BGM‐treated inhibited these changes. Figure 1C showed that BGM treatment reduced the Pb content in the kidney. Kidney injury indicators showed that BGM improved kidney function, which downregulated the levels of creatinine, urea and uric acid (Figure 1D). Figure 1E showed that Pb increased albumin level and decreased globulin level and total protein level in serum; BGM‐treated mitigated these changes. The changes in kidney damage markers are shown in Figure 1F; BGM‐treated suppressed the mRNA expressions of Kim‐1, Ngal, Col1A1, and Tgf‐β. On these indicators, we found that a high concentration of BGM (0.22 mg/kg) showed better protective effects than 0.05 mg/kg. These results demonstrated that BGM alleviated Pb‐caused kidney injury. Figure 1G showed that the liver tissue in the control group appears normal. Hepatocytes are well arranged in cords around the central vein. There are no noticeable pathological changes such as inflammation, necrosis, or fibrosis. Sinusoids are clearly visible, and no abnormalities in cellular structure can be observed. The Pb‐treated group shows significant pathological changes compared to the control. Hepatocytes exhibit signs of cellular swelling, indicating possible hydropic degeneration. There is congestion in the sinusoids and central veins. Some necrotic cells are visible, with nuclei showing pyknosis (condensed nuclei), which is a sign of irreversible cell damage. Inflammatory cell infiltration is evident, suggesting a response to lead‐induced injury. The liver tissue in the Pb + BGM group shows a notable reduction in the pathological features compared to the lead‐treated group. Hepatocyte architecture appears partially restored. There is less congestion and fewer necrotic cells compared to the Pb group. However, mild vacuolization and residual inflammation can still be observed, indicating that the damage has been mitigated but not fully reversed.

### 3.2 BGM Improves Pb‐Caused Oxidative Stress and Inflammation in Mouse Kidney

**FIGURE 2:**
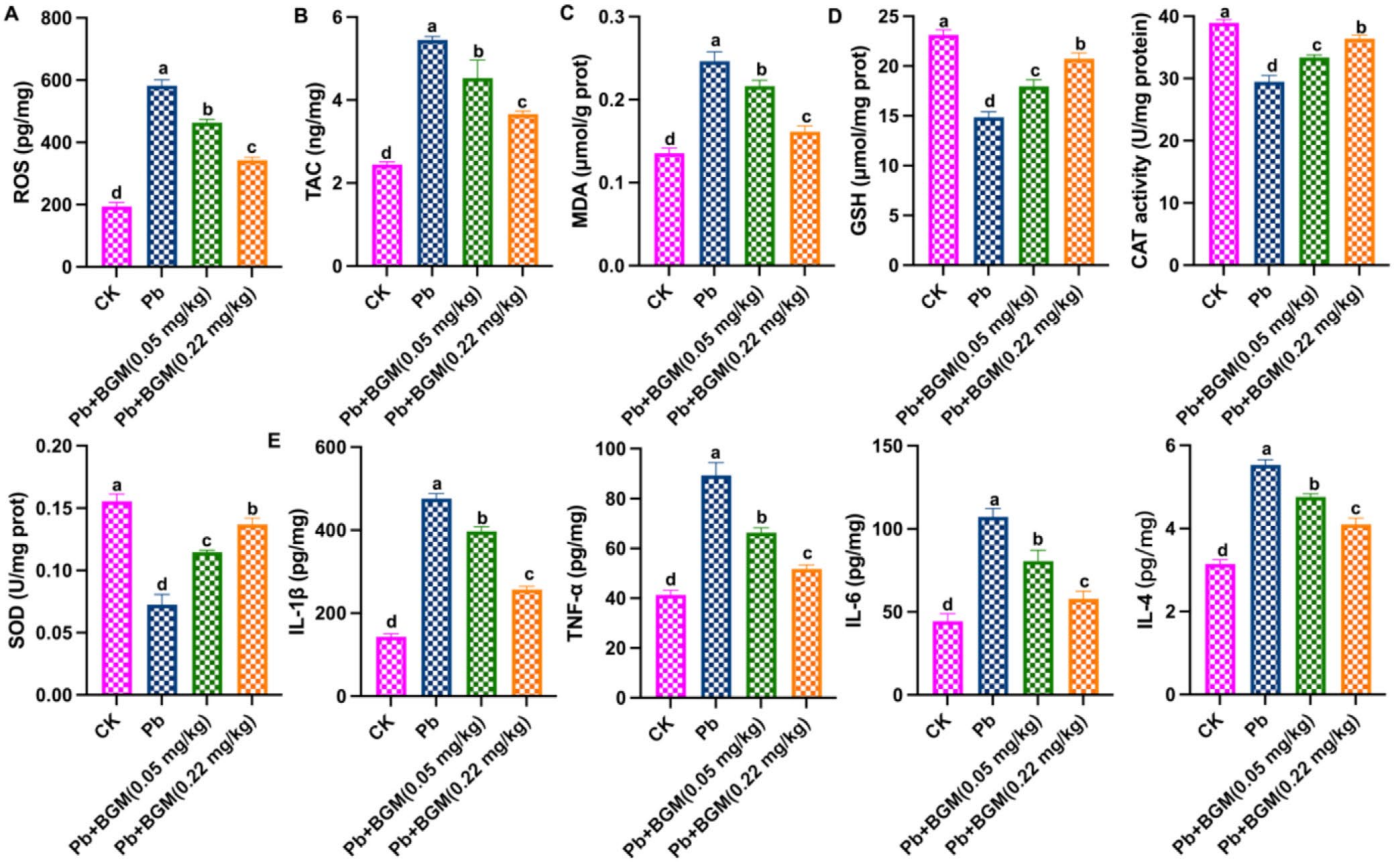
BGM improves Pb‐caused oxidative stress and inflammation in the mouse kidney. (A) ROS level; (B) TAC content; (C) MDA content; (D) Levels of SOD, CAT, and GSH; (E) Levels of IL‐6, IL‐1β, IL‐4, and TNF‐α. Different letters indicate significant differences (*p* < 0.05).

As shown in Figure 2A–C Pb exposure promoted ROS accumulation and increased TAC and MDA contents, while BGM treatment decreased these levels. Figure 2D showed the changes in antioxidant enzyme activities and inflammation‐related factors; Pb exposure down‐regulated the levels of SOD, CAT, and GSH, and up‐regulated the levels of IL‐6, IL‐1β, IL‐4, and TNF‐α, and BGM alleviated these changes. These results indicated that BGM improves Pb‐caused oxidative stress and inflammation.

### 3.3 BGM Improves Pb‐Caused Pyroptosis in Mouse Kidney

**FIGURE 3:**
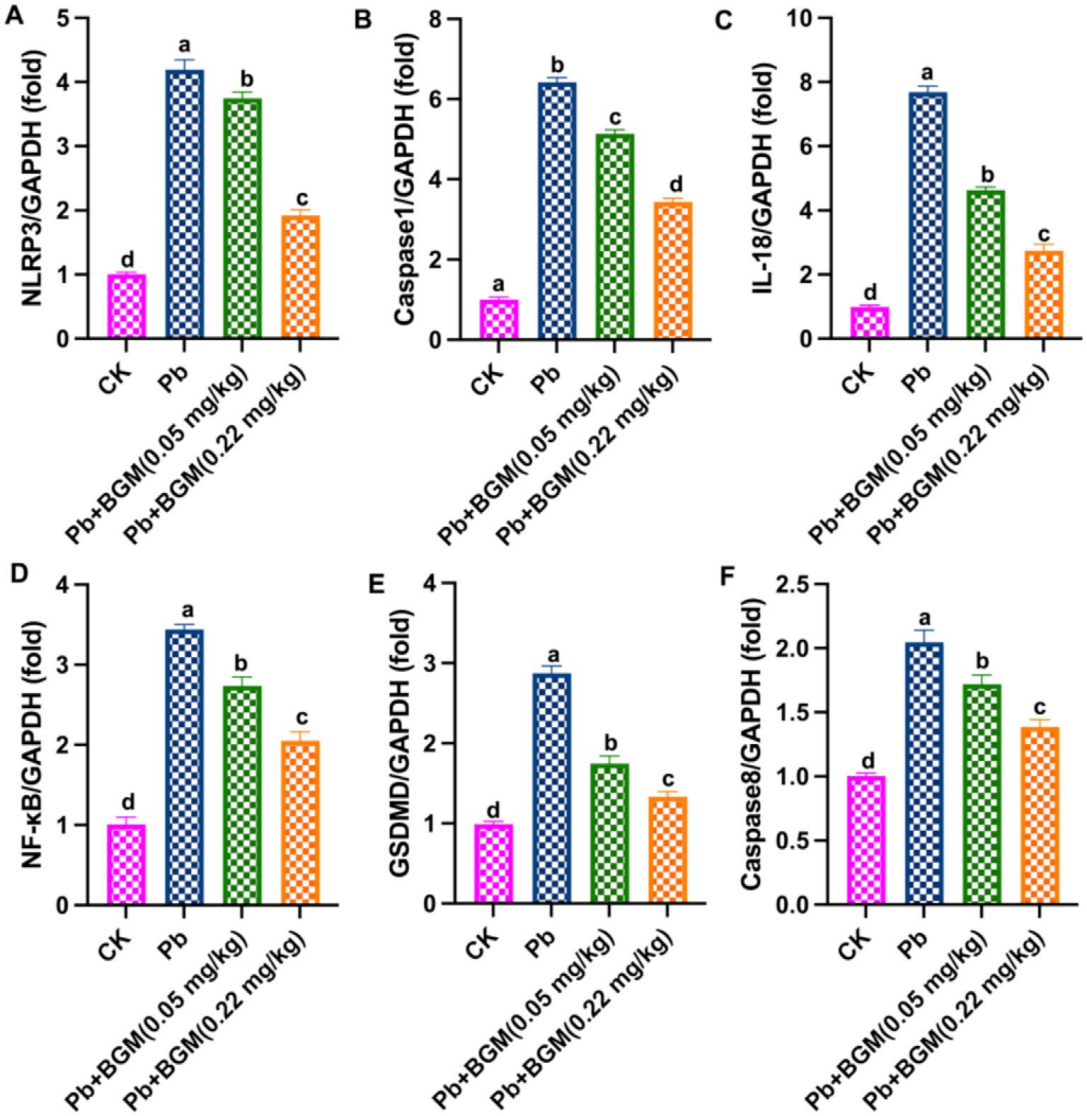
BGM improves Pb‐caused pyroptosis in the mouse kidney. (A) NLRP3 mRNA level; (B) caspase‐1 mRNA level; (C) IL‐18 mRNA level; (D) NF‐κB mRNA level; (E) GSDMD mRNA level; (F) caspase‐8 mRNA level. Different letters mean significant differences (*p* < 0.05).

Figure 3 shows the mRNA expressions of pyroptosis‐related mediators. Pb increased the levels of NLRP3, caspase‐1, IL‐18, NF‐κB, GSDMD, and caspase‐8, while BGM decreased these levels, which indicated that BGM improved Pb‐caused pyroptosis in the mouse kidney.

### 3.4 TLR2 is the Key Regulator of BGM Protects Against Pb‐Induced Kidney Injury

**FIGURE 4:**
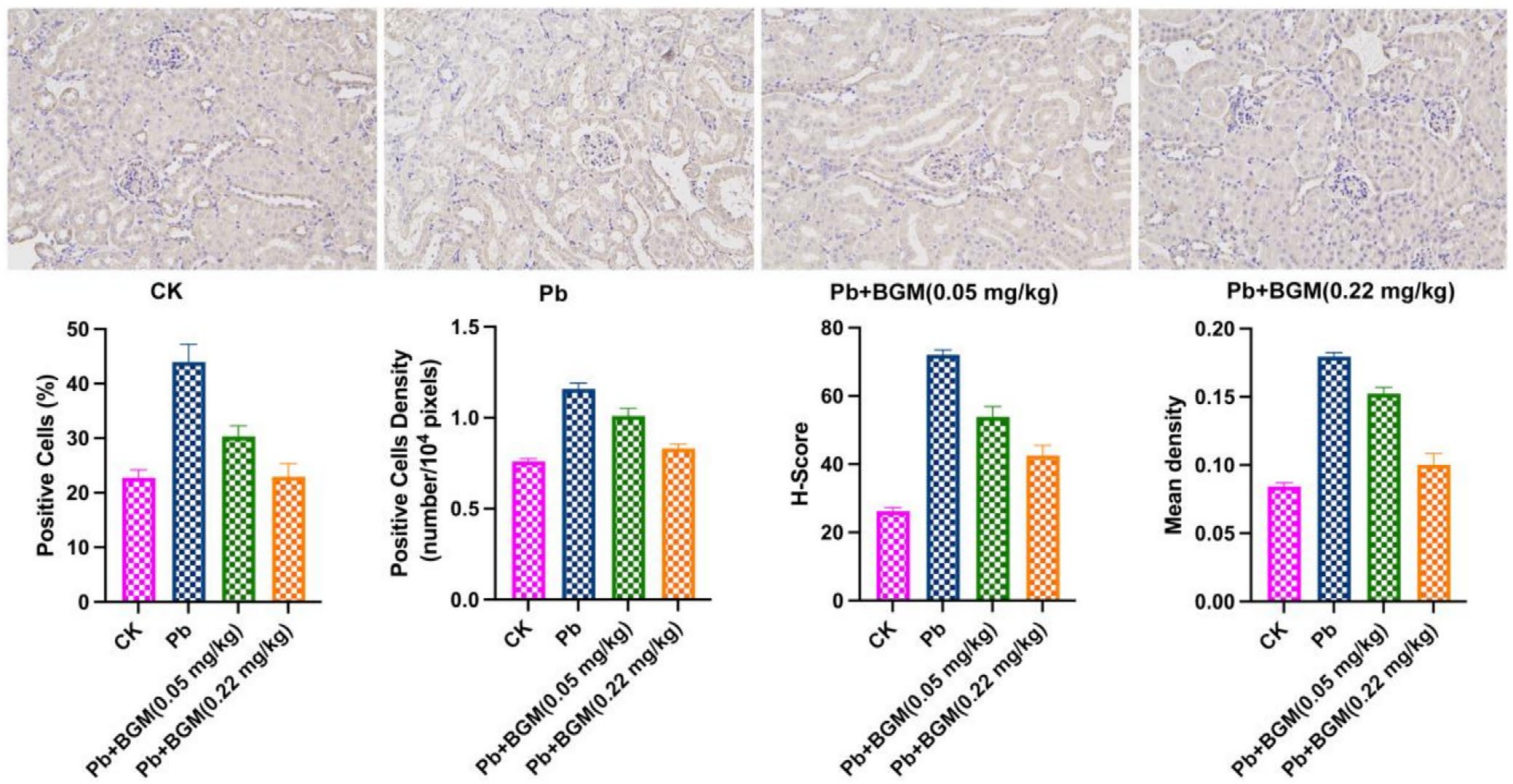
Immunohistochemistry staining of TLR2. Different letters mean significant differences (*p* < 0.05).

TLR2 may serve as a key regulatory factor in the protective effects of BGM against Pb‐induced kidney injury. In this study, TLR2 expression levels in the kidneys of mice from different treatment groups were assessed via immunohistochemistry, as shown in Figure 4. Pb exposure significantly elevated TLR2 levels in the kidneys. However, BGM treatment effectively reduced TLR2 expression, including the number of positive cells, cell density, H‐score, and mean TLR2 density, with the 0.22 mg/kg dose showing a more pronounced effect. These findings suggest that BGM mitigates Pb‐induced kidney damage by inhibiting the TLR2 upregulation caused by Pb exposure. Thus, TLR2 is a key regulatory factor mediating the protective effects of BGM.

### 3.5 Silencing of TLR2 Enhanced the Protective Effect of BGM on Pb‐Induced Damage in HK‐2 Cells

**FIGURE 5:**
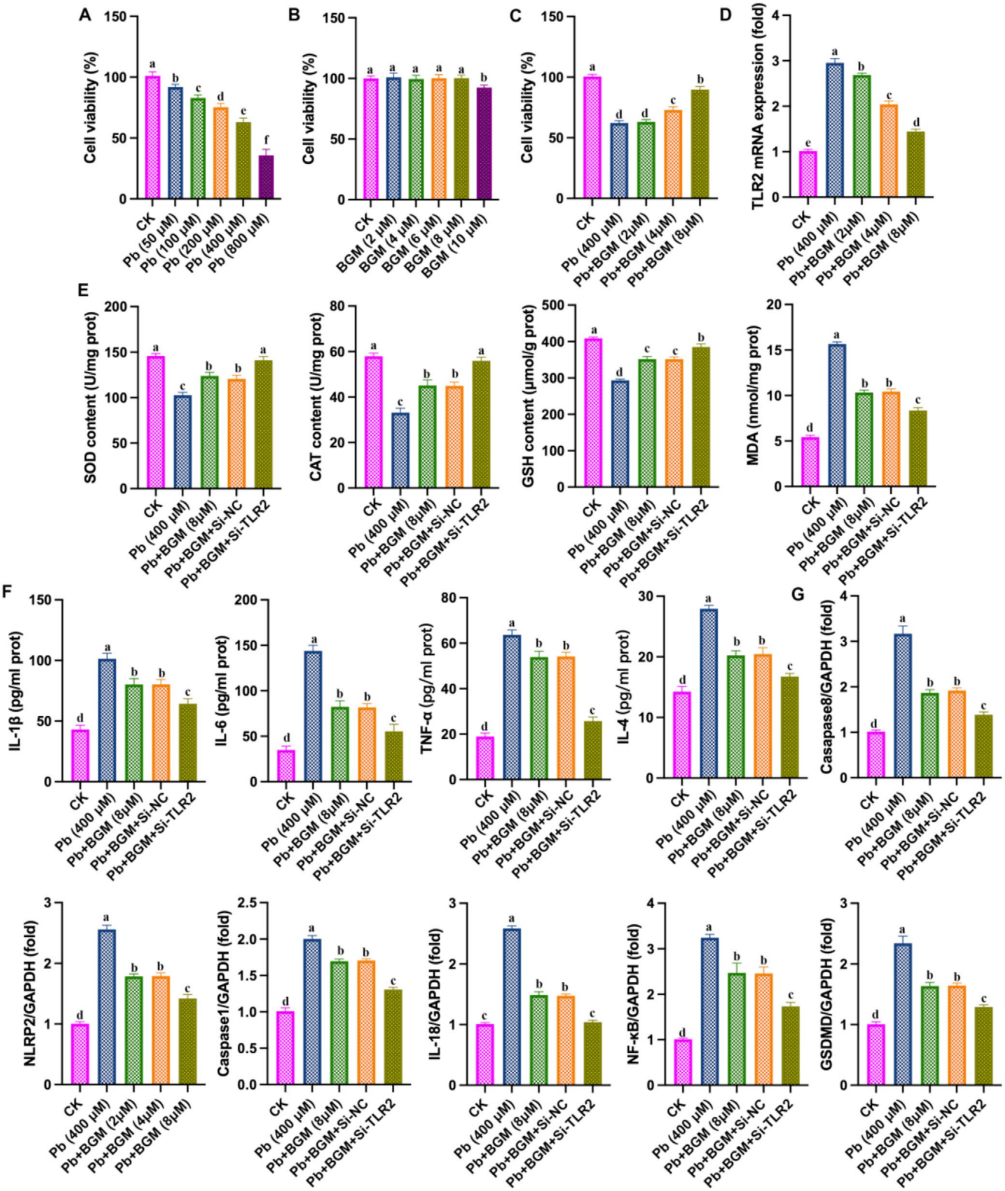
Silencing of TLR2 enhanced the protective effect of BGM on Pb‐caused injury in HK‐2 cells. (A–C) Cell viability; (D) TLR2 mRNA level; (E) levels of SOD, CAT, and GSH; (F) IL‐6, IL‐1β, IL‐4, and TNF‐α; (G) mRNA levels of NLRP3, caspase‐1, IL‐18, NF‐κB, GSDMD, and caspase‐8.

To further explore the role of TLR2 in BGM protecting against Pb‐induced damage, we silenced TLR2 in Pb‐induced damage in HK‐2 cells. As shown in Figure 5A, Pb treatment decreased HK‐2 cell viability in a dose‐dependent manner (50–800 μM), and the cell viability is about 65% at 400 μM compared to the control; thus, we selected 400 μM for the subsequent experiments. Figure 5B showed that BGM at 4, 6, 8, and 10 μM had no effect on cell viability. Figure 5C,D showed that BGM (4 μM and 8 μM) increased HK‐2 cell viability and BGM (2, 4, and 8 μM) decreased TLR2 mRNA expression, and 8 μM of BGM showed the best protective effect; thus, we selected the dose of 8 μM BGM for the subsequent experiments. Figure 5E–G showed that silencing TLR2 increased the levels of SOD, GPx, and CAT, and decreased the levels of MDA, TNF‐α, IL‐1β, IL‐6, IFNβ, NLRP3, caspase‐1, IL‐18, NF‐κB, GSDMD, and caspase‐8, compared to BGM‐treated. These results demonstrated that silencing TLR2 enhanced the protective effect of BGM against Pb‐induced injury in HK‐2 cells, which inhibited oxidative stress, inflammation, and pyroptosis.

### 3.6 Overexpressing of TLR2 Weakened the Protective Effect of BGM on Pb‐Induced Damage in HK‐2 Cells

**FIGURE 6:**
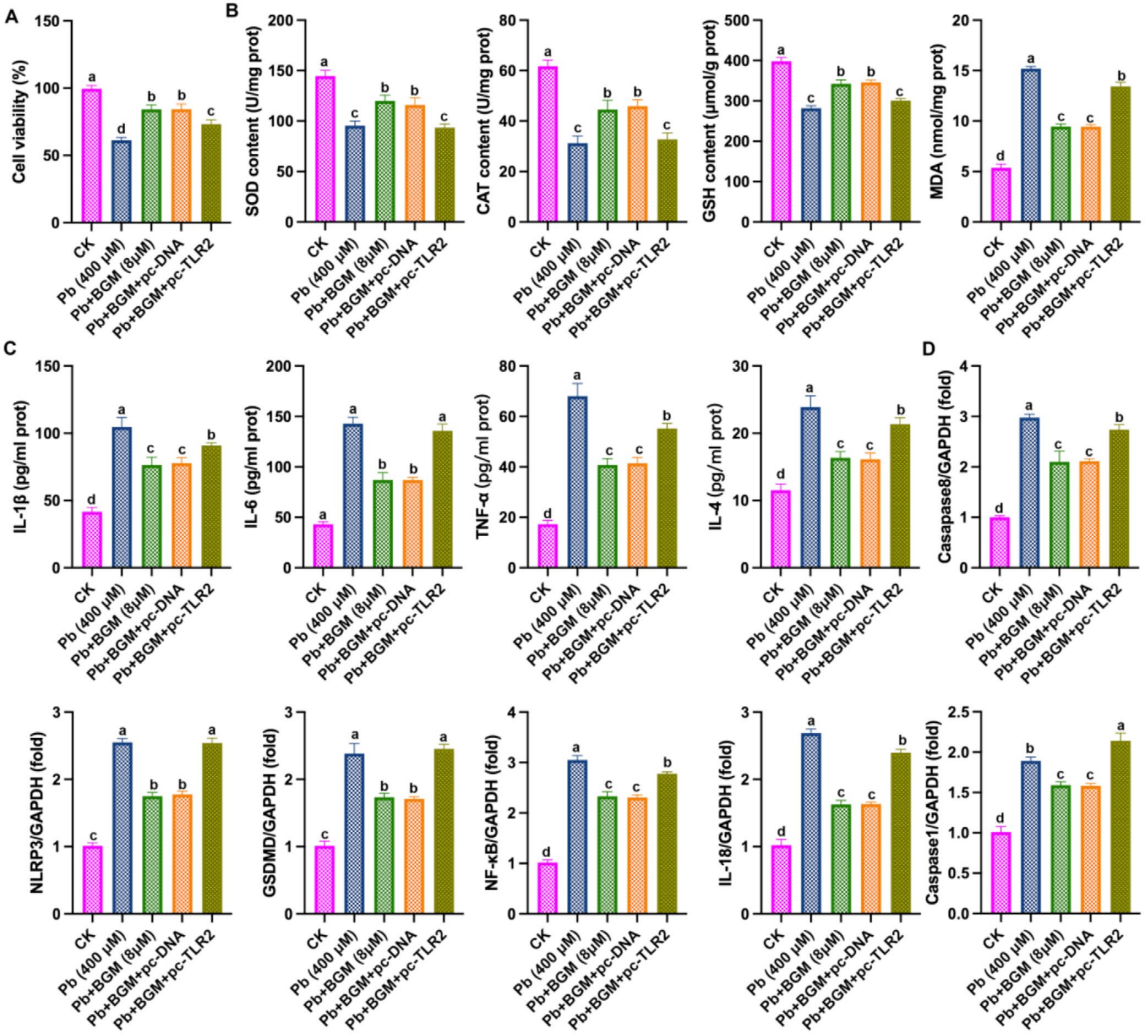
Overexpression of TLR2 weakened the protective effect of BGM on Pb‐caused injury in HK‐2 cells. (A) Cell viability; (B) Levels of SOD, CAT, and GSH; (C) IL‐6, IL‐1β, IL‐4, and TNF‐α; (D) mRNA levels of NLRP3, caspase‐1, IL‐18, NF‐κB, GSDMD, and caspase‐8. Different letters indicated significant differences (*p* < 0.05).

To further explore the role of TLR2 in BGM protecting against Pb‐induced damage, we overexpressed TLR2 in HK‐2 cells. As shown in Figure 6A, overexpressing TLR2 reduced HK‐2 cell viability compared to BGM‐treated cells. Figure 6B–D showed that overexpressing TLR2 reduced the levels of SOD, GPx, and CAT, and up‐regulated the levels of MDA, TNF‐α, IL‐1β, IL‐6, IFNβ, NLRP3, caspase‐1, IL‐18, NF‐κB, GSDMD, and caspase‐8, compared to BGM‐treated. These results demonstrated that overexpression of TLR2 weakened the protective effect of BGM against Pb‐induced damage in HK‐2 cells, which promoted oxidative stress, inflammation, and pyroptosis.

## 4 Discussion

This study aimed to explore the protective effects of BGM, a natural furanocoumarin found in citrus fruits, on lead (Pb)‐induced kidney damage in mice. The findings highlight the efficacy of BGM in mitigating Pb‐induced renal dysfunction, oxidative stress, inflammation, and pyroptosis, contributing to the development of potential therapeutic strategies for Pb‐induced toxicity.

Pb exposure caused significant renal impairment, as evidenced by elevated levels of creatinine, urea, and uric acid, which are established markers of kidney dysfunction. Pb accumulates in the kidneys, disrupting cellular homeostasis and inducing oxidative stress, ultimately leading to nephrotoxicity (Lian et al. 2023; Miao, Miao, Shi, et al. 2022; Song and Zhu 2020). BGM treatment, especially at a higher dose (0.22 mg/kg), significantly ameliorated these dysfunction markers, highlighting its therapeutic potential in mitigating Pb‐induced renal damage. These findings are consistent with previous studies, which emphasize BGM's antioxidative and anti‐inflammatory properties in various tissue injury models, further supporting its potential as an effective agent against heavy metal‐induced nephrotoxicity (Liu et al. 2022; Wang et al. 2022; Yazıcı et al. 2023).

Oxidative stress is a well‐established mechanism of Pb‐induced toxicity, primarily driven by the overproduction of ROS and the depletion of antioxidant defenses (Park et al. 2020; Shilpa et al. 2021). In our study, Pb exposure resulted in elevated levels of MDA, a lipid peroxidation marker, and reduced activities of key antioxidant enzymes such as SOD and CAT. This oxidative imbalance contributes to cellular damage and organ dysfunction. BGM treatment significantly restored the activities of SOD and CAT, while reducing MDA levels in kidney tissues, indicating that BGM exerts a potent antioxidant effect. This is consistent with previous studies that have shown the antioxidant capacity of BGM in other models of oxidative stress, such as in cafeteria diet‐induced liver injury (Yazıcı et al. 2023). The ability of BGM to restore antioxidant defenses likely plays a critical role in mitigating Pb‐induced kidney damage.

In addition to oxidative stress, Pb‐induced toxicity is closely associated with inflammation and pyroptosis, a form of programmed cell death that is mediated by the inflammasome and contributes to tissue injury (Miao, Miao, Teng, and Xu 2022). Our results revealed that Pb exposure triggered an upregulation of inflammatory markers such as IL‐6, IL‐1β, and TNF‐α, as well as key components of the pyroptosis pathway, including NLRP3, caspase‐1, and GSDMD. These findings are consistent with previous reports linking Pb‐induced damage to the activation of inflammatory pathways (Albarakati et al. 2020). Importantly, BGM significantly reduced the expression of these inflammatory and pyroptotic markers, indicating that BGM not only reduces oxidative stress but also attenuates the downstream inflammatory and pyroptotic responses. Furthermore, the suppression of NLRP3 inflammasome activation by BGM is particularly noteworthy, as this pathway has been identified as a central mediator of Pb‐induced inflammation and pyroptosis (Wei et al. 2021). In line with these findings, studies have demonstrated that inhibiting the NLRP3 pathway can reduce inflammation and tissue damage in Pb‐induced injury models. BGM's capacity to modulate this pathway further reinforces its potential therapeutic application in heavy metal toxicity.

In this study, we identified TLR2 as a critical regulatory factor in Pb‐induced kidney injury, with BGM exerting its protective effects primarily through the modulation of the TLR2/NF‐κB signaling axis. TLR2, a key receptor in the innate immune response, is well documented for its role in recognizing PAMPs and DAMPs, which can activate downstream inflammatory pathways (Apaydın et al. 2016; Naghib et al. 2022). Pb exposure can mimic DAMPs, leading to the overactivation of TLR2 and subsequent inflammatory cascades, thereby contributing to oxidative stress, inflammation, and pyroptosis in the kidneys (Mabrouk et al. 2016). Our results demonstrated that Pb significantly upregulated TLR2 expression in renal tissues, which was strongly correlated with increased levels of pro‐inflammatory cytokines, along with the activation of NF‐κB.

BGM treatment, especially at higher doses, markedly downregulated TLR2 expression, as observed in the immunohistochemical analysis. This reduction in TLR2 was accompanied by decreased activation of NF‐κB and lower levels of inflammatory cytokines, indicating that BGM effectively suppressed the TLR2‐mediated inflammatory response. By inhibiting the TLR2/NF‐κB axis, BGM may have also mitigated the activation of the NLRP3 inflammasome, as evidenced by the reduced expression of NLRP3, caspase‐1, and GSDMD in the kidney tissue of BGM‐treated mice. This suggests that TLR2 plays a pivotal role not only in initiating inflammation but also in promoting pyroptosis, a form of programmed cell death associated with inflammation. Moreover, the antioxidant effects of BGM, as shown by the restoration of SOD and CAT activities and reduction in MDA levels, further support the notion that BGM's inhibition of TLR2 may also help alleviate oxidative stress. It is well known that oxidative stress and inflammation are closely linked, and the suppression of TLR2 likely plays a dual role in attenuating both processes.

Furthermore, this study found that overexpression of TLR2 further amplified the activation of NF‐κB and increased the levels of these cytokines, suggesting that heightened TLR2 activity intensifies the inflammatory response induced by Pb. Additionally, TLR2 overexpression was associated with elevated oxidative stress markers, such as MDA, and increased activation of pyroptosis‐related proteins, including NLRP3, caspase‐1, and GSDMD. Conversely, when TLR2 was silenced, the protective effects of BGM were significantly enhanced. Silencing TLR2 not only reduced NF‐κB activation and pro‐inflammatory cytokine levels but also attenuated oxidative stress and pyroptosis. This was evidenced by the decreased expression of NLRP3, caspase‐1, and GSDMD, as well as the restoration of antioxidant enzyme activities. These findings indicate that TLR2 is a critical mediator of Pb‐induced kidney damage and that BGM's protective effects are largely dependent on its ability to downregulate TLR2 expression.

In summary, our findings highlight TLR2 as a key regulatory factor in Pb‐induced kidney damage and suggest that BGM protects renal tissues by targeting the TLR2/NF‐κB signaling pathway. This mechanism underscores the importance of TLR2 in mediating Pb toxicity and opens up potential avenues for therapeutic interventions targeting TLR2 in heavy metal‐induced nephrotoxicity.

## 5 Conclusion

**FIGURE 7:**
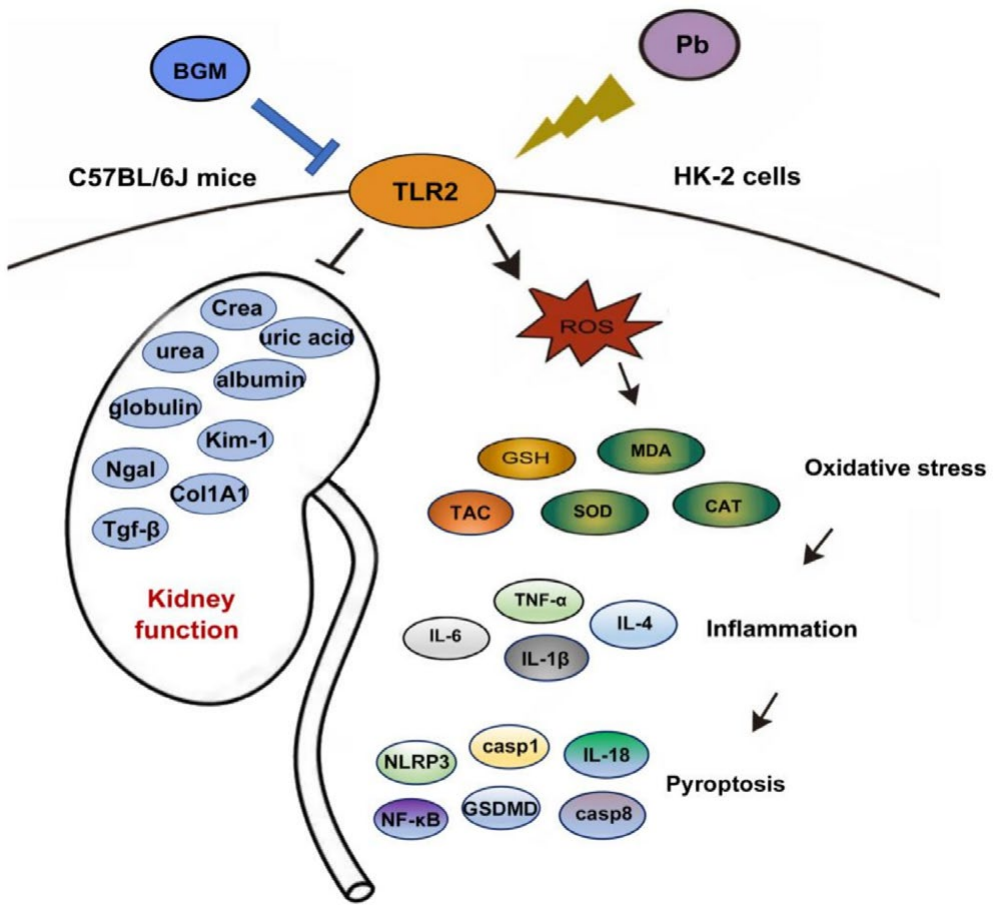
BGM mitigated Pb‐caused kidney damage through TLR2‐mediated oxidative stress, inflammation, and pyroptosis.

This study demonstrates that BGM significantly protects against Pb‐induced kidney damage by modulating the TLR2/NF‐κB signaling axis. The results reveal that Pb exposure leads to increased TLR2 expression, which triggers inflammatory and pyroptotic responses, as well as oxidative stress in the kidneys (Figure 7). BGM treatment effectively downregulated TLR2, leading to the suppression of the NF‐κB pathway and its associated inflammatory markers. This not only reduced inflammation and pyroptosis but also alleviated oxidative stress, highlighting the multifaceted protective effects of BGM. Our findings identify TLR2 as a critical factor in Pb‐induced renal injury and suggest that targeting this receptor could be a promising therapeutic strategy for managing heavy metal toxicity. The ability of BGM to modulate TLR2 expression and downstream signaling provides new insights into its protective mechanism, paving the way for further research into the therapeutic applications of plant‐derived bioactive compounds.

## Author Contributions

**Yanping Zhu:** data curation (equal), formal analysis (equal), investigation (equal), writing – original draft (equal). **Kaisheng Zhang:** software (equal), supervision (equal), validation (equal), writing – original draft (equal). **Song Wen:** data curation (equal), formal analysis (equal), software (equal), supervision (equal), validation (equal).

## Ethics Statement

The authors have nothing to report.

## Conflicts of Interest

The authors declare no conflicts of interest.

## Data Availability

All data generated during this study are included in this article.
